# Ligand–Receptor Interaction Combined with Histopathology Improves Glioma Prognostic Model

**DOI:** 10.3390/biomedicines14051110

**Published:** 2026-05-14

**Authors:** Lun Gao, Rui Zhang, Xiaonan Zhu, Haitao Xu, Qianxue Chen, Min Peng, Junhui Liu

**Affiliations:** 1 Department of Neurosurgery, Renmin Hospital of Wuhan University, Wuhan 430060, China; lungao@whu.edu.cn (L.G.);; 2 Department of Neurosurgery, The First People’s Hospital of Yunnan Province, The Affiliated Hospital of Kunming University of Science and Technology, Kunming 650032, China; 3 Department of Oncology, Renmin Hospital of Wuhan University, Wuhan 430060, China; ricky815@126.com

**Keywords:** glioma, ligand–receptor interaction, deep learning, spatial transcriptomics, prognosis, pathomics

## Abstract

**Background:** Glioblastoma (GBM) is the most aggressive primary brain tumor with extremely poor prognosis. Conventional diagnostic and prognostic approaches remain inadequate, highlighting the need for integrative strategies to improve patient outcomes. **Methods:** We analyzed ligand–receptor (L–R) interactions in TCGA-GBM transcriptomes using BulkSignaL-R, and validated their spatial expression patterns with single-cell RNA sequencing (scRNA-seq) and spatial transcriptomics datasets. Prognostic histopathological features were extracted from hematoxylin and eosin (H&E)-stained sections through omics-guided feature identification, followed by classification using machine learning algorithms. **Results:** We identified four pivotal L–R pairs (LTB–CD40, VEGFA–ITGB1, FN1–COL13A1, and TGM2–ITGB1) to construct a risk model, which served as an independent prognostic factor for overall survival. The multivariate Cox regression analyses revealed that the risk score was significantly associated with Overall Survival (OS) (HR = 1.67, 95% CI: 1.25–2.25, *p* < 0.001). High-risk patients exhibited distinct molecular signatures, including CALN1 mutations, specific CNV patterns, and enriched Notch/interferon-γ signalings. scRNA-seq and spatial transcriptomics revealed that these L–R pairs were predominantly expressed in gMES-like glioma cells, OPC-like cells, and pericytes. Finally, our deep learning model successfully stratified risk groups based on histological features, identifying specific tumor regions (Clusters 0, 2, 4, and 5) as critical determinants of prognosis (AUC = 0.750 by Logistic Regression). **Conclusions:** We developed a novel multi-modal framework integrating L–R interactomics and deep learning-based pathomics. This approach not only elucidates the molecular and spatial landscape of glioma intercellular communication but also provides a methodological framework for risk stratification.

## 1. Introduction

Gliomas constitute the most prevalent primary brain tumors, representing approximately 80% of malignant central nervous system neoplasms. Among these, high-grade gliomas—particularly glioblastoma (GBM)—carry a notoriously poor prognosis. Despite multimodal therapies encompassing surgical resection, radiotherapy, and chemotherapy, median overall survival for GBM remains below 15 months [1]. These tumors are hallmarked by pronounced spatial heterogeneity and pervasive invasiveness; tumor cells infiltrate along white matter tracts and vasculature, complicating complete resection and contributing to recurrence and therapeutic resistance [2]. Consequently, early detection and precise molecular stratification are indispensable to enhancing patient outcomes. Yet, due to challenges in acquiring representative brain tissue and the extreme intratumoral heterogeneity, conventional diagnostic modalities remain inherently constrained.

Emerging evidence underscores the critical role of intercellular communication within the tumor microenvironment (TME) in glioma pathogenesis. Ligand–receptor interactions serve as pivotal mediators of cross-talk between neoplastic cells and cellular constituents of the TME—such as immune infiltrates, astrocytes, and endothelial cells—thereby modulating processes including tumor-associated immunosuppression, angiogenesis, and invasive dissemination. These signaling networks correlate strongly with clinical outcomes and present promising avenues for biomarker discovery and therapeutic targeting [3,4]. Single-cell transcriptomic studies have systematically identified key prognostic ligand–receptor interactions, including autocrine pairs within neoplastic cells and communication networks between cancer stem-like cells and macrophages, demonstrating the utility of such signatures in survival prediction models [5,6,7].

Hematoxylin-eosin (HE) staining, the cornerstone of histopathological evaluation, excels in revealing tissue architecture and morphological nuances but falls short in capturing underlying molecular signatures. In recent years, convolutional neural networks (CNNs) and other deep learning methods applied to high-throughput HE image analysis have been successfully employed in tumor classification, prognosis assessment, and molecular feature prediction [8,9]. Moreover, multimodal frameworks combining histopathological imaging with multi-dimensional omics data have shown improved prognostic power. For example, an integrated model leveraging histopathological image features and multi-omics data—including genomics, transcriptomics, and proteomics—achieved enhanced prediction of molecular subtypes and survival in glioblastoma [10]. Other multimodal deep learning strategies integrating histology, MRI, genomic and clinical data similarly outperformed unimodal models, with C-indices reaching ~0.79 versus ~0.72 in conventional settings [11,12].

The prognostic gap addressed in our model lies in the profound intra-tumoral heterogeneity and adaptive metabolic plasticity of GBM, which current WHO-integrated classifications (e.g., IDH status, MGMT methylation) do not fully capture. In this study, we propose a machine learning framework that integrates image analysis and multi-omics features to construct a ligand–receptor risk model based on HE-stained sections for predicting clinical outcomes in glioma patients. This approach integrates three key dimensions—tissue architecture, spatial localization, and molecular interactions—thereby significantly improving predictive accuracy compared with traditional models. Moreover, this work aims to identify key ligand–receptor signaling pathways and their associated cell types in glioma, providing theoretical insights and methodological support for the development of precision therapeutic strategies.

## 2. Materials and Methods

### 2.1. Data Collection and Preprocessing

We downloaded and organized the raw count matrix of GBM samples from the GDC data center (including 160 type 01A primary tumor samples) [13], and filtered the expression data for protein-coding genes based on the GENCODE v38 annotation file^1. Samples with <10 million mapped reads or >20% ribosomal RNA content were excluded; all 160 primary tumor (01A) samples passed these criteria. The flowchart of the research and data processing was presented as Figure S1.

### 2.2. Ligand–Receptor (L–R) Pair Identification

The R package BulkSignalR (v4.1.2) was employed to analyze the transcriptomic expression profiles of TCGA-GBM [14]. The specific steps were as follows:

(1) Data Preparation and Normalization: The prepareDataset function was used to normalize the tumor sample expression matrix (with the method set to Total Count, TC), and genes supporting at least 80 ligand–receptor pairs were selected as input;
(2) Parameter Learning and Model Construction: The learnParameters function was applied to estimate the covariate distribution in the background model and output the fitness scores for each regulatory model;
(3) Interaction Scoring and Pathway Reduction: The initialInference function was utilized to preliminarily infer all possible ligand–receptor pairs and their significance. Significant L–R pairs were mapped to known cell communication pathways, and redundancy was removed using the reduceToBestPathway function, retaining only the ligand–receptor pairs associated with the most significantly optimal pathways.

### 2.3. Construction and Validation of Prognostic Signature

Univariate Cox regression analysis was performed to determine the hazard ratios (HR) and prognostic significance of differentially expressed genes, with genes exhibiting *p* < 0.05 selected as prognosis-related L–R feature pairs. Based on these prognosis-related feature pairs, LASSO regression was applied to identify feature pairs with non-zero regression coefficients (using the R package glmnet, v4.1-4). Concurrently, a random forest model was used to calculate feature importance scores, and the top 10 important features were selected. The intersection of features identified by LASSO and random forest was then taken, and a multivariate Cox regression model was constructed based on the selected feature pairs to calculate the risk score for each sample: (Surv(OS.time, OS) ~ Σ L–R).

### 2.4. Mutation Analysis

Based on the SNV data from the TCGA-GBM cohort [15], the maftools package(v2.14.0) was used to construct a MAF object and generate an oncoplot depicting the top 20 high-frequency mutated genes^5.

To investigate differences in genomic copy number variations (CNVs) between high- and low-risk group cancer samples, the TCGAbiolinks package (v2.26.3) was employed to download Copy Number Segment data for the TCGA-GBM project from the GDC database, with only tumor tissue samples selected. The copy number segment data were preprocessed, including removal of mitochondrial chromosome (chrM) segments and elimination of redundant segments to ensure accuracy and consistency. The GISTIC2.0.23 tool was applied to perform CNV region analysis separately for the high- and low-risk groups, identifying broad amplification and deletion regions and generating genome-wide G-scores. Finally, the readGistic and gisticChromPlot functions from the maftools package(v2.26.3) were used to read the GISTIC output files and visualize the results^6.

To evaluate differences in somatic mutation signatures between risk groups, single-base substitution (SBS) signature fitting analysis was conducted based on the COSMIC (Catalogue of Somatic Mutations in Cancer) mutation signature database. Somatic mutation information for each sample was extracted from MAF-formatted mutation files and converted to VCF format, followed by import as a GRanges object list. Using the mut_matrix function from the MutationalPatterns package(v3.8.1), a 96-channel base mutation matrix was constructed based on the imported VCF list and the reference genome (BSgenome.Hsapiens.UCSC.hg38). This matrix quantified the frequency distribution of different types of single-base substitutions (e.g., C>T, G>A) in trinucleotide contexts for each sample. The mutation spectrum of each sample was fitted to known COSMIC SBS mutation signatures to obtain the contribution weights of each sample across different signatures (contribution matrix).

### 2.5. Single-Cell Data Download and Preprocessing

Single-cell data from GBM patients (GSE271959) were obtained from the GEO database (https://www.ncbi.nlm.nih.gov/geo (accessed on 1 September 2025)) [16]. The R package Seurat was used for downstream analysis of the 10× count matrix [17]. To exclude low-quality cells and lowly expressed genes, the following thresholds were applied:

(1) Number of features per cell between 500 and 5000;
(2) Number of counts per cell between 500 and 20,000;
(3) Mitochondrial gene percentage per cell less than 20%.

The NormalizeData function was used to normalize the scRNA-seq dataset, and the FindVariableFeatures function with the “vst” method was employed to identify 3000 highly variable genes. Data scaling was performed using the ScaleData function, and principal component analysis (PCA) was conducted with the RunPCA function. The number of PCs was selected via visualization using the ElbowPlot function. Dimensionality reduction and visualization were achieved with the RunUMAP function, setting n.neighbors to 20 and dims to 1:20. The Harmony algorithm was applied to remove batch effects across different samples by integrating single-cell RNA sequencing data from multiple samples and adjusting cell expression profiles for consistency, thereby eliminating batch effects. Batch correction was performed using the R package Harmony (v1.2.0) to avoid interference in downstream analyses. The FindNeighbors function was used with the top 20 principal components to construct a shared nearest neighbor (SNN) graph, followed by cell clustering using the FindClusters function with resolution set to 0.3. Differentially expressed genes between cell types (i.e., marker genes for each cell type) were identified using the FindAllMarkers function in Seurat, with min.pct = 0.25, logfc.threshold = 0.5, and retention of genes with *p* < 0.05.

### 2.6. Spatial Transcriptomics Single-Cell Data Download and Preprocessing

Spatial transcriptomics data for glioma were downloaded from the GEO database: GSE194329, including 5 glioma samples (GSM5833533–GSM5833537). Quality control and alignment of raw spatial transcriptomics sequencing data were performed using spaceranger-2.1.1. The gene-spot matrix generated after processing ST sample data with Seurat was normalized for raw counts across different spots using the SCTransform function. Using the cell annotations and corresponding gene expression profiles from single-cell analysis as reference, RCTD was applied to deconvolve the spatial transcriptomics data, assigning mixed cell types to spatial transcriptomics spots in doublet mode^10.

### 2.7. Pathological Histological Analysis

Data Acquisition and Preprocessing: The study data were sourced from the TCGA-GBM database, comprising 860 whole slide images (WSIs) from 389 glioma patients. In the preprocessing stage, each WSI was precisely segmented into 256 × 256 pixel patches, with background regions removed, resulting in 803,803 patches for subsequent analysis. To reduce color variations across slides, Macenko’s method was applied for color normalization, and Z-score standardization was performed on the RGB channels to ensure data consistency.

Deep Learning Feature Extraction and Clustering Analysis: The deep learning model ResNet-50 was utilized to extract 2048-dimensional deep features from the average pooling layer of each patch. These high-dimensional features were then reduced to 32 dimensions via principal component analysis (PCA) to decrease computational complexity while preserving key information. Subsequently, K-means clustering was applied to divide the patches into 6 categories (k = 6). Prior to selecting this parameter, we evaluated multiple values of k (from 3 to 10) using the elbow method (inertia) and silhouette scores to determine the optimal number of clusters. K = 6 provided the optimal balance. Clustering results were visualized using t-SNE and annotated by experienced pathologists based on histological features (e.g., tumor regions, stroma, muscle, invasive fronts). Finally, these cluster distributions were mapped back to the original WSIs to intuitively display the spatial distribution of different tissue regions^11.

Pathological Region Selection Strategy: To optimize model performance, a pathological region selection strategy was implemented. Patient data were randomly divided into training and testing sets at a 0.75:0.25 ratio (with random seed set to 42 for reproducibility), ensuring the split was at the patient level. On the training set, a VGG16 convolutional neural network (CNN) was trained. VGG16 was trained for 50 epochs (early stopping patience = 10) with Adam optimizer (learning rate = 1 × 10^−4^, weight decay = 1 × 10^−5^), batch size = 32, and binary cross-entropy loss. Data augmentation (random rotation, flipping, and color jitter) was applied during training. On the testing set, model performance was evaluated for each of the 6 clusters. Based on pathologists’ expert opinions and analyses of model performance and tissue distribution, clusters highly relevant to tumors and adjacent stroma were retained (excluding lower-performing Clusters 1 and 3). A majority voting approach was adopted to aggregate predictions from high-performance cluster patches, generating cluster-based WSI-level classifications.

Multiple Instance Learning (MIL) and WSI-Level Prediction: To transition from patch-level to WSI-level predictions, two multiple instance learning (MIL) aggregation strategies were employed: Patch Likelihood Histogram (PLH), which discretizes patch prediction probabilities and constructs histograms to capture the prediction distribution across the entire WSI; and Bag of Words (BoW), which converts patch prediction probabilities into feature vectors using TF-IDF (term frequency-inverse document frequency) for subsequent machine learning model training. A total of 45 features were extracted from these methods. Feature selection was performed using Pearson correlation coefficients to eliminate highly collinear features. Finally, random forest (RF) and logistic regression (LR) machine learning models were trained on the selected features, with performance evaluated via 3-fold cross-validation, ultimately generating WSI risk prediction results.

### 2.8. Statistical Analysis

Statistical processing relied on R software (v4.1.2). We applied the Wilcoxon rank-sum test for two-group comparisons and the Kruskal–Wallis test for multi-group analysis. Survival outcomes were evaluated using Kaplan–Meier curves and log-rank tests. Significance levels are indicated as * *p* < 0.05, ** *p* < 0.01, *** *p* < 0.001, and **** *p* < 0.0001.

## 3. Results

### 3.1. Ligand–Receptor-Based Prognostic Risk Model for Glioma

**Figure 1:**
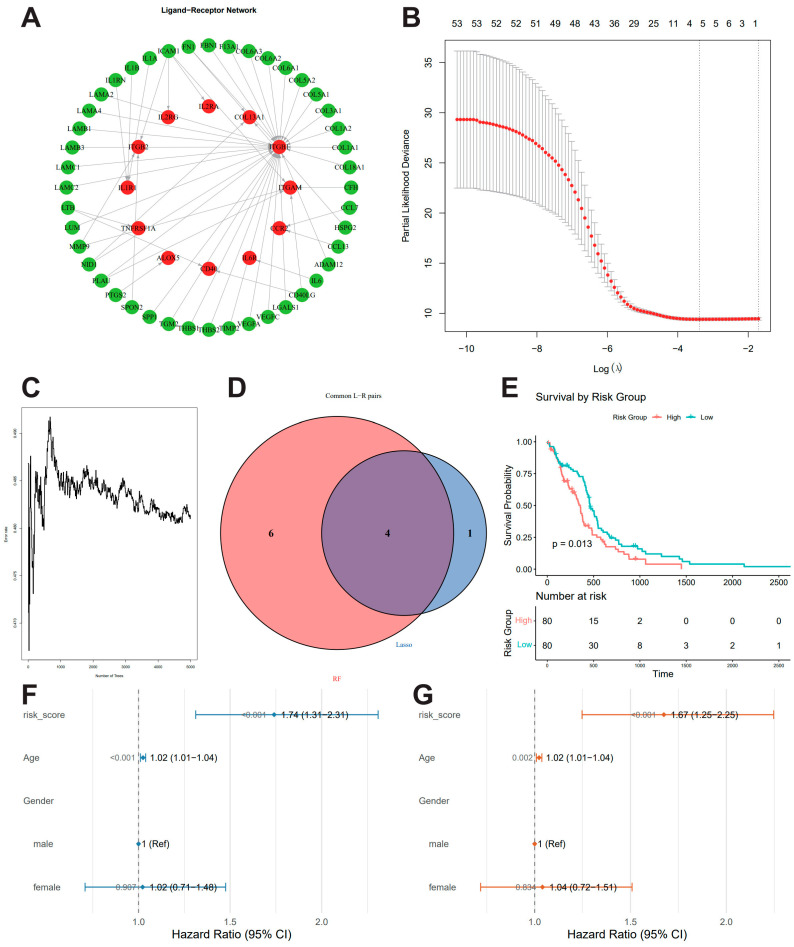
**Identification and construction of the ligand–receptor (L–R)-based prognostic risk model.** (**A**) Network visualization of significant ligand–receptor interactions associated with patient prognosis. Ligands are represented as green nodes and receptors as red nodes. (**B**) Selection of optimal parameters for the LASSO-Cox regression model. (**C**) Variable importance of L–R pairs derived from the Random Survival Forest (RSF) model. (**D**) Venn diagram illustrating the intersection of candidate L–R pairs identified by both LASSO-Cox and Random Forest algorithms. (**E**) Kaplan–Meier survival analysis comparing overall survival (OS) between the high-risk and low-risk groups. (**F**,**G**) Univariate (**F**) and multivariate (**G**) Cox proportional hazards regression analyses evaluating the association between the constructed L–R risk signature and patient prognosis, adjusted for other clinical covariates. The transcriptomic expression profiles were obtained from TCGA-GBM.

We applied the BulkSignaL–R package (v4.1.2) to analyze the transcriptomic expression matrix of TCGA-GBM samples and identified 191 significant ligand–receptor interaction pairs (q < 1 × 10^−8^) [13]. To further evaluate the activity and prognostic significance of these ligand–receptor (L–R) signaling axes at the individual-patient level, we calculated pathway-specific L–R feature scores for each sample. Subsequently, we constructed univariate Cox proportional hazards regression models for each L–R feature score and applied Benjamini–Hochberg (BH) correction to control the false discovery rate, yielding 53 survival-associated L–R pairs (Figure S2). A network diagram of these survivaL–Related ligand–receptor interactions was generated (Figure 1A), in which ligand genes are represented as green nodes and receptor genes as red nodes.

To further refine representative prognostic features from this network, we employed both LASSO-Cox regression and random forest models. In the LASSO-Cox model, the optimal penalty parameter λ was selected using 10-fold cross-validation, resulting in a fitted model that retained 5 non-zero coefficient L–R pairs deemed important prognostic features (Figure 1B, Figures S3 and S4). In parallel, we constructed a random survival forest model using the randomForestSRC package with 5000 trees, extracting the variable importance scores of each feature (Figure 1C). The integration of both approaches yielded a final subset of 4 key L–R pairs (Figure 1D): LTB–CD40, VEGFA–ITGB1, FN1–COL13A1, and TGM2–ITGB1.

Based on these selected features, we extracted corresponding activity scores and overall survival (OS, OS.time) data to build a multivariate Cox proportional hazards regression model. Patients were categorized into high- or low-risk cohorts based on the median risk score. Subsequent analysis confirmed that the high-risk group experienced significantly reduced overall survival (Figure 1E).

To further assess whether the constructed prognostic model served as an independent prognostic factor in the clinical context, univariate Cox regression analyses were performed. The results demonstrated that the risk score was significantly associated with OS (HR = 1.74, 95% CI: 1.31–2.31, *p* < 0.001; Figure 1F). After adjusting for clinical variables such as age and gender, multivariate Cox regression analyses confirmed that the risk score retained independent prognostic value (Figure 1G). These findings suggest that the proposed risk model provides a robust and independent predictor of survival risk across diverse clinical contexts, underscoring its potential utility in clinical applications.

### 3.2. Molecular Characteristics of Different Risk Groups and Potential Therapeutic Drugs

**Figure 2:**
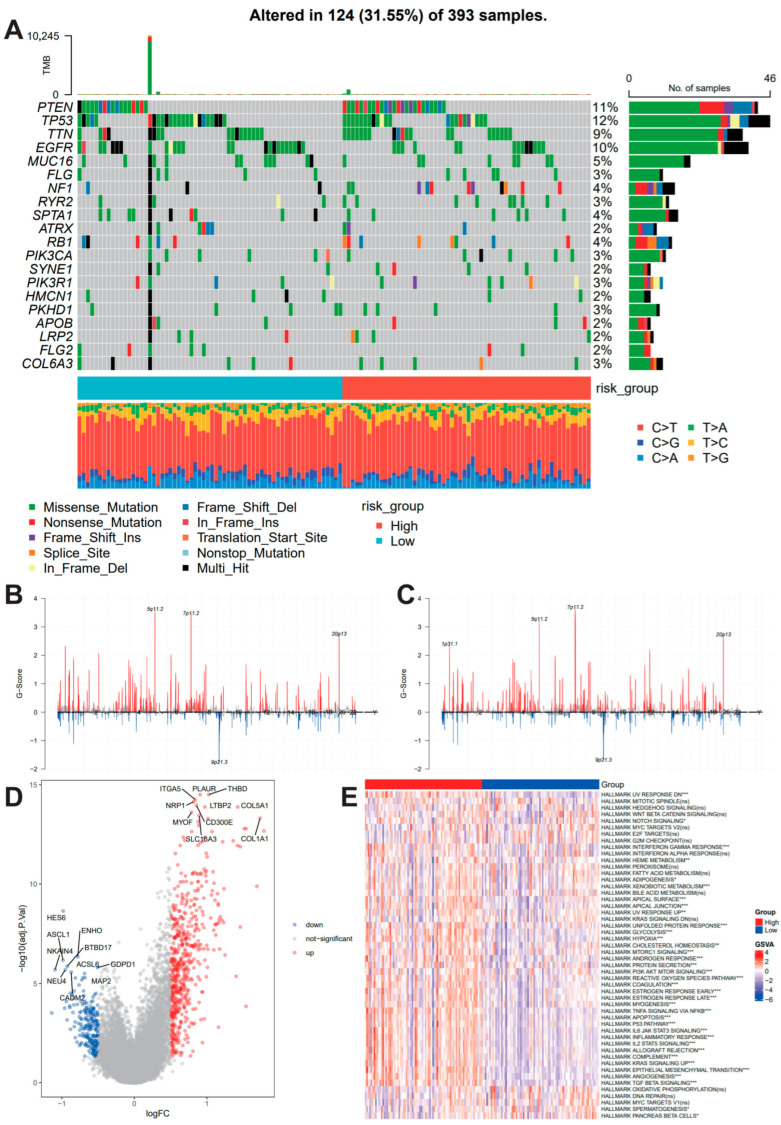
**Multi-omics landscape characterization of high- and low-risk groups.** (**A**) Mutational landscape of glioma patients. Top bar plot: Tumor Mutational Burden (TMB) frequency. Central waterfall plot: Somatic mutation distribution of the top mutated genes. Right bar plot: Mutation counts per gene. Bottom strip: Risk group classification (Red: High-risk; Blue: Low-risk). The SNV data were obtained from the TCGA-GBM cohort. (**B**,**C**) Genome-wide Copy Number Variation (CNV) profiles, including amplification and deletion frequencies, in the high-risk (**B**) and low-risk (**C**) groups. (**D**) Volcano plot depicting differentially expressed genes (DEGs) between the high- and low-risk groups. (**E**) Gene Set Enrichment Analysis (GSEA) highlighting significantly enriched Hallmark pathways in different risk populations. ns, no significance, * *p* < 0.05, ** *p* < 0.01, *** *p* < 0.001.

To gain deeper insights into the molecular dynamics of different prognostic subtypes, we conducted multi-omics analyses. Gene mutation analysis revealed that PTEN, TP53, and TTN were the most frequently mutated genes in cancer patients (Figure 2A). Comparing the mutation frequencies of genes in high-risk and low-risk groups revealed that CALN1 was more frequently mutated in the high-risk group (Figure S5), suggesting that its mutation status may be associated with poor prognosis.

In addition to gene mutations, copy number variation is also a hallmark of tumor genomic mutations. To assess differences in somatic mutation signatures between different risk groups, we downloaded copy number segment data from the TCGA database and selected data from tumor tissues. The copy number segment data were then preprocessed, including removing mitochondrial chromosomes (chrM) and removing redundant segments to ensure the accuracy and consistency of the segment information. Using the GISTIC2 tool, CNV regions were analyzed for both high-risk and low-risk groups, identifying regions of widespread amplification and deletion, and generating genome-wide G-scores. Finally, the readGistic and gisticChromPlot functions in the maftools package were used to read the GISTIC output files and plot the genome-wide copy number variation distribution for the high-risk and low-risk groups, reflecting the differences in amplification and deletion characteristics at the chromosomal level (Figure 2B,C).

In addition to genomic data, we also analyzed transcriptomic data from cancer patients. Using DESeq2, we performed differential gene expression analysis between high-risk and low-risk cancer patients (Figure 2D). GSEA highlighted distinct pathway variations, including Notch and interferon-γ signaling, between the risk groups (Figure 2E). Additionally, oncoPredict screening pinpointed 10 candidate drugs with elevated IC50 values in high-risk tumors (Figure S6), suggesting potential therapeutic relevance. These drugs may be potential therapeutic candidates identified in silico, which need experimental validation.

### 3.3. Cellular and Spatial Distribution of Prognosis-Related Ligand–Receptor Pairs

**Figure 3:**
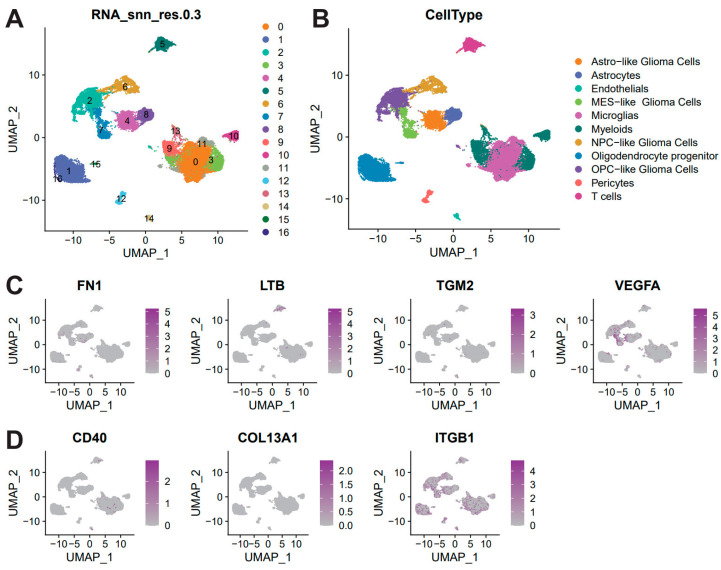
**Single-cell transcriptomic analysis of prognostic L–R pair expression.** (**A**) Uniform Manifold Approximation and Projection (UMAP) plot visualizing the clustering of single cells. (**B**) Dot plot illustrating the expression of canonical marker genes used for cell type annotation. (**C**,**D**) Expression distribution of the identified key ligand (**C**) and receptor (**D**) genes across malignant cell subclusters.

To investigate the cellular distribution of prognosis-related ligand–receptor (L–R) pairs, we analyzed single-cell RNA-sequencing (scRNA-seq) data from glioma patients (GSE271959). Following data quality control, we employed UMAP for cell type clustering, identifying 17 distinct cell clusters at a resolution of 0.3 (Figure 3A). Subsequently, marker gene identification was performed for these cell clusters (Figure S7). A total of 11 major cell types were ultimately identified (Figure 3B). Expression analysis was conducted for the four L–R pairs previously identified (Figure 3C,D). Notably, the VEGFA-ITGB1 exhibited significantly elevated expression in gMES-like Glioma Cells, OPC-like Glioma Cells, Myeloids and T cells.

**Figure 4:**
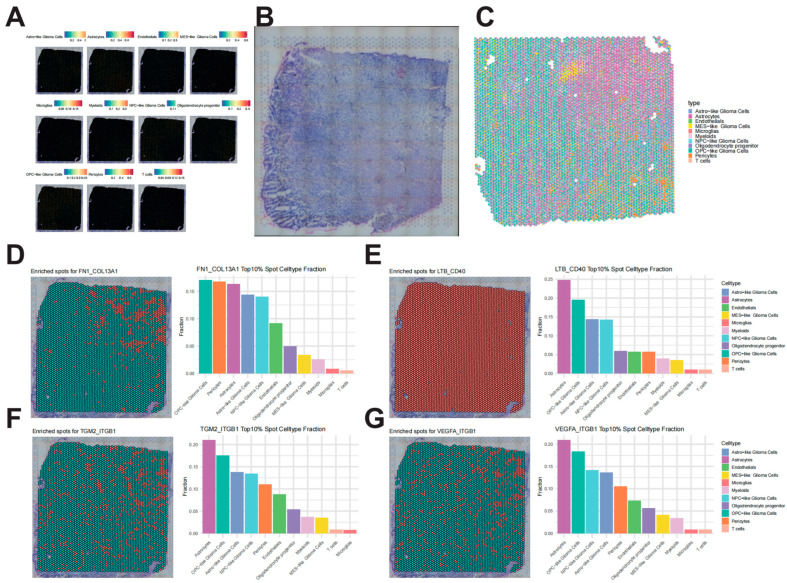
**Spatial co-localization and distribution of L–R pairs using spatial transcriptomics.** (**A**) Spatial deconvolution mapping of distinct cell types onto tissue spots, revealing the cellular architecture within the tumor microenvironment. (**B**) The corresponding Hematoxylin and Eosin (H&E) stained tissue section. (**C**) Heatmap showing the enrichment proportion of different cell types across spatial spots. (**D**–**G**) Spatial expression and co-localization patterns of key L–R pairs. **Left:** Scatter plots visualizing the spatial distribution, where red dots indicate regions enriched for both ligand and receptor, and green dots indicate non-enriched regions. **Right:** Histograms quantifying the expression proportion of L–R pairs across different cell types.

To explore the spatial distribution of prognosis-related L–R pairs, spatial single-cell transcriptomic data from five samples were analyzed for spatial localization. Cell types identified from scRNA-seq were projected onto tissue spots to reveal their spatial organization within the tumor (Figure 4A–C). Subsequent analysis of the spatial distribution of the identified L–R pairs demonstrated that different L–R pairs were highly expressed in different cell types (Figure 4D–G), mainly consistent with the results from single-cell analysis. FN1_COL13A1 L–R pair was mainly expressed in OPC-like glioma cells and pericytes. While for other 3 L-4 pairs, they were mostly expressed in astrocytes and OPC-like glioma cells.

### 3.4. Histology-Based Classification of High-Risk Gliomas

**Figure 5:**
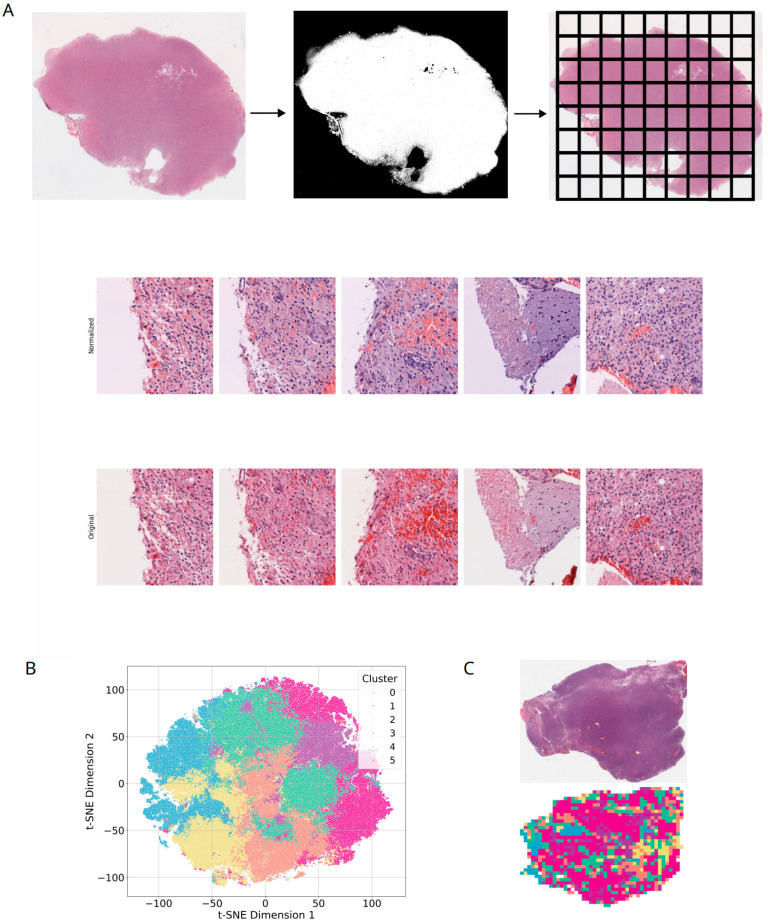
**Deep learning-based histological clustering and spatial visualization of H&E images.** (**A**) Workflow for pathological image analysis. The pipeline includes tissue boundary detection, tiling into fixed-size patches, and color normalization to mitigate batch effects. (**B**) t-SNE visualization of image patches clustered by K-means based on deep features extracted via ResNet50. (**C**) Spatial reconstruction of the histological clusters on the whole-slide images (WSIs), mapping the distribution of different morphological patterns.

Building on the systematic analysis of ligand–receptor interactions and their cellular distribution, we sought to establish a robust histology-driven classification strategy using hematoxylin–eosin (HE) stained slides from TCGA-GBM. A total of 860 whole-slide images were collected (Figure 5A). Following tissue boundary detection and tiling into 256 × 256-pixel patches, background regions were excluded, yielding 803,803 image patches. To minimize inter-slide variability, color normalization was performed using the Macenko method, and RGB channels were standardized by Z-score transformation (Figure 5A).

Deep feature extraction was conducted using ResNet50, generating 2048-dimensional features that were subsequently reduced to 32 principal components via PCA. K-means clustering (k = 6) identified six distinct patch categories, which were visualized using t-SNE and mapped back onto the WSIs to reveal their spatial distribution (Figure 5B,C).

### 3.5. Deep Learning Validation of Tumor Subtypes via Pathological Regions

**Figure 6:**
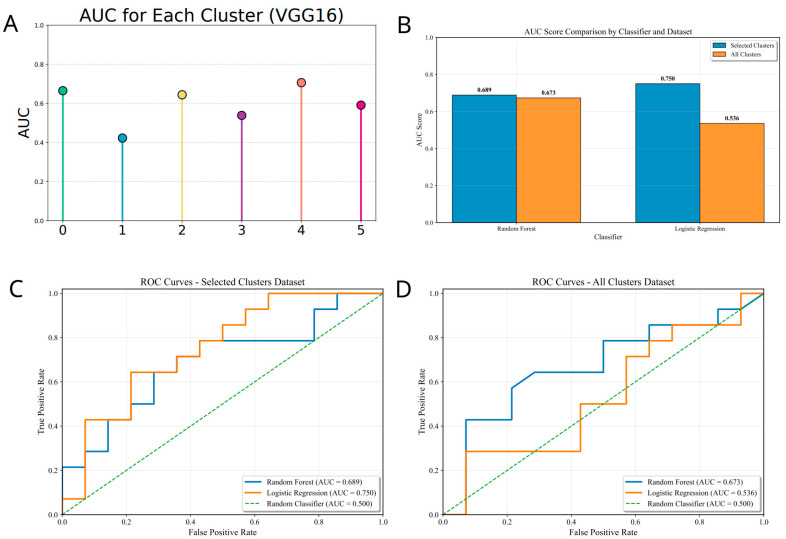
**Performance evaluation of the weakly supervised learning models.** (**A**) Bar chart comparing the Area Under the Curve (AUC) of prediction models constructed using different feature sets. (**B**,**C**) Performance comparison of Random Forest (**B**) and Logistic Regression (**C**) classifiers. (**D**) ROC curves comparing the AUC of all clusters dataset.The curves demonstrate the model accuracy using feature-selected datasets versus unselected datasets.

Patients were randomly assigned to training and testing cohorts at a 3:1 ratio (seed = 42), ensuring patient-level independence. A VGG16-based CNN classifier was trained to stratify tumors, with Grad-CAM employed to highlight histological regions driving predictions. These saliency maps were projected back onto WSIs, delineating the spatial distribution of high- and low-risk areas. Model performance was evaluated across all six clusters in the test cohort (Figure 6A).

Integrating pathologists’ insights on tissue morphology with cluster-level performance, two poorly performing clusters (Clusters 1 and 3) were excluded. The remaining clusters were aggregated into WSI-level predictions using two complementary strategies: Patch Likelihood Histograms (PLH), which capture the distribution of prediction probabilities, and Bag-of-Words (BoW), which convert patch probabilities into feature vectors through TF-IDF weighting. After collinearity reduction via Pearson correlation, 45 representative features were retained for classifier construction. Random forest and logistic regression models were trained with three-fold cross-validation, both achieving higher accuracy once low-performance clusters were excluded (Figure 6C).

**Figure 7:**
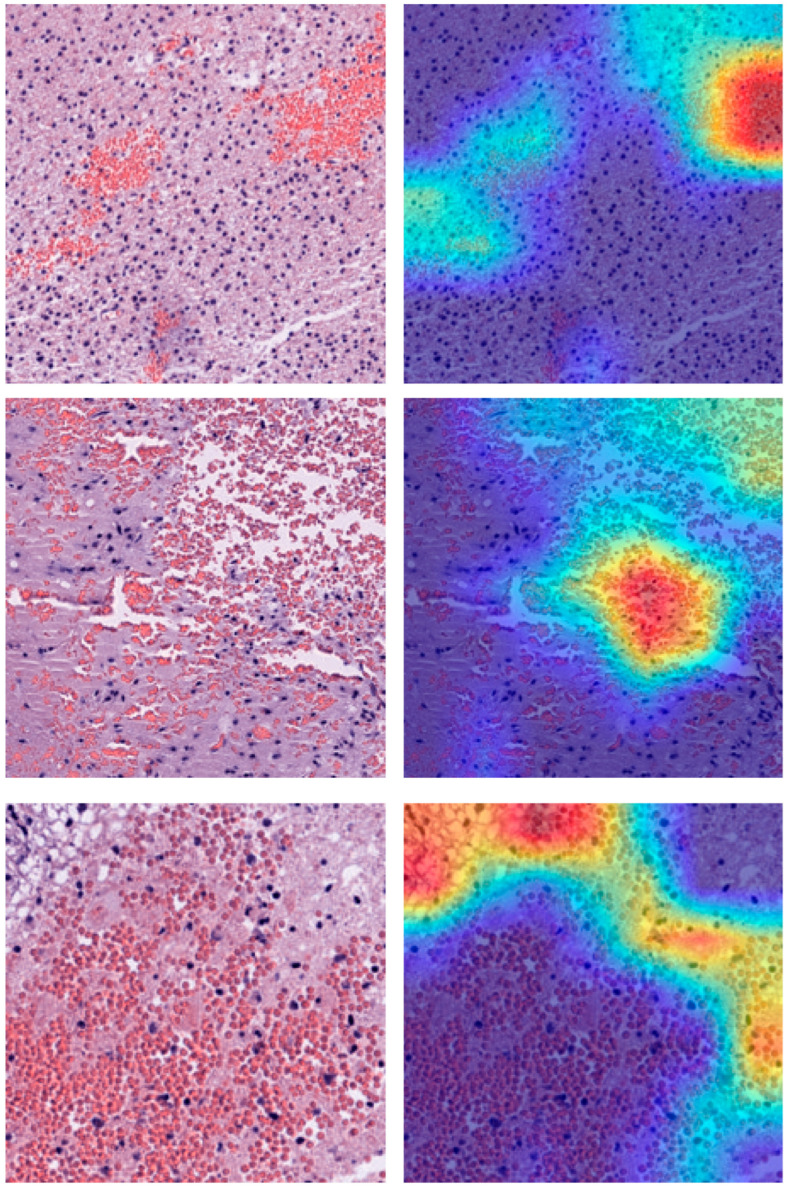
**Visualization of high-risk histological features using Gradient-weighted Class Activation Mapping (Grad-CAM).** Representative H&E images and their corresponding deep learning attention maps. **Left (Original):** Original tissue patches. **Right (High Risk):** Saliency maps overlaid on the original images. The heatmaps (generated via Grad-CAM) highlight the regions of interest (ROI) driven by the deep learning model, indicating histological features most predictive of high risk.

Collectively, we identified Clusters 0, 2, 4, and 5 as the critical determinants of patient risk. Heatmap visualization further revealed that these high-risk regions were predominantly localized within tumor parenchyma, underscoring their biological relevance and predictive potential (Figure 7).

## 4. Discussion

Our study presents a novel multi-modal prognostic framework in glioma that integrates multi-omics, single-cell RNA sequencing (scRNA-seq), spatial transcriptomics, and deep learning analysis of HE-stained histopathology. The key merits of our model are: (1) identification of prognostic ligand–receptor (L–R) pairs, (2) classification of patients into high- and low-risk groups based on survival outcomes, and (3) development of a clinically accessible deep learning model that identifies six histopathological clusters—especially clusters 0, 2, 4, and 5—that effectively stratify patient risk. This model stratifies patients into high- and low-risk groups based on survival data, while a complementary deep learning framework applied to H&E-stained WSIs identifies critical tissue clusters (0, 2, 4, and 5) with superior predictive utility. The application of a pathology region selection strategy further refines model performance, enabling precise differentiation of risk phenotypes and automating ROI delineation. These discoveries underscore the model’s potential to improve clinical prognosis prediction in glioma.

In recent years, multi-omics sequencing of large samples (including transcriptome, proteomics, metabolome, etc.) has been used to stratify the prognostic risk of different glioma patients, reflecting the powerful ability of multi-omics to predict patient prognosis [18,19,20]. For example, a multimodal fusion framework combining radiological imaging, pathology, genomics, transcriptomics, and proteomics uncovered novel subtypes with enhanced prognostic ability [21]. This study demonstrated how radiomic features from MRI scans, when fused with histopathological image features (HIF), predicted molecular characteristics and overall survival. Meanwhile, AI-driven multi-omics approaches in glioblastoma have employed single-cell and spatial omics with radiomics to model intra-tumoral heterogeneity [22]. These integrations often relied on machine learning to correlate imaging phenotypes with genomic alterations, such as IDH mutations or MGMT promoter methylation, which are critical for glioma classification. However, our model distinguished itself by emphasizing deep learning on H&E-stained whole-slide images (WSIs) to identify specific histopathological clusters (0, 2, 4, and 5), which was better than traditional markers in risk stratification [23]. Unlike studies that primarily fuse bulk multi-omics with imaging, we incorporated spatial transcriptomics to map gene expression patterns onto these clusters, enabling a spatially resolved prognosis that captures dynamic TME interactions. This addressed a key gap in prior research, where histopathological analysis was often qualitative or limited to basic feature extraction, rather than automated cluster-based prediction.

The incorporation of multi-omics and spatial transcriptomic data enhanced our biological understanding of molecular features influencing glioma prognosis. Similar integrative approaches have illuminated grade-related gene networks and uncovered hundreds of prognostic genes beyond traditional markers like IDH1 or PTEN [24,25].

Meanwhile, single-cell and spatial omics continued to uncover context-dependent L–R signaling pathways, such as myeloid–tumor interactions mediated via specialized pairs [26]. Our model elucidated the intricate interplay within the glioma tumor microenvironment (TME), where L–R signaling drove immunosuppression, angiogenesis, and invasion—hallmarks of poor prognosis. For instance, VEGFA-ITGB1 interactions likely promoted vascular remodeling and tumor dissemination, consistent with prior observations of VEGF-mediated endothelial crosstalk in GBM. Similarly, LTB-CD40 might amplify inflammatory responses via lymphoid tissue organizer cells, while FN1-COL13A1 and TGM2-ITGB1 could facilitate extracellular matrix remodeling, fostering therapeutic resistance [27].

Recent multi-omics studies across solid tumours demonstrated that ligand–receptor (L–R) interactions serve as robust prognostic biomarkers. In hepatocellular carcinoma and lung adenocarcinoma, ligand–receptor (L–R)-based risk models integrating transcriptomics and spatial data identified signaling networks driving angiogenesis and immune evasion [28,29]. Similarly, colorectal and head-and-neck cancers have used L–R scoring combined with digital pathology to define prognostic subtypes [30]. However, these models relied mainly on bulk or single-cell transcriptomics and lacked spatial or histological validation. Our study advances this field by coupling L–R pair discovery with deep learning on whole-slide H&E images, achieving spatially resolved risk prediction. Unlike previous frameworks that correlated gene expression with limited imaging features [8,25,31], our model captured tumor–astrocyte signaling axes (LTB–CD40, VEGFA–ITGB1, FN1–COL13A, TGM2–ITGB1) and mapped their histological localization. This integration enables cross-scale interpretability, linking cellular communication to tissue-level morphology—an advantage over existing radiogenomic or transcriptomic models.

The advantages of our risk model lie in its multimodal integration, which significantly enhanced interpretability compared to previous studies. For instance, building upon a deep autoencoder-based multi-omics model for glioma subtype classification, we achieved high accuracy by employing dimensionality reduction techniques analogous to principal component analysis (PCA) and clustering steps. Furthermore, our integration of spatial and histopathological layers refined the characterization of tumor microenvironment (TME) dynamics. By incorporating ligand–receptor (L–R) pairs that mediate intercellular communication within the TME, the model identified potential signaling patterns associated with tumor progression and immune evasion. Targets such as VEGFA-ITGB1 have been previously validated for their therapeutic potential in prior research, and our model extends these findings by statistically linking such pairs with prognostic risk stratification in multi-omics datasets [32,33]. We optimized prior workflows through a pathology region selection strategy that automates region-of-interest (ROI) delineation, potentially reducing reliance on manual pathologist intervention. Of course, this study still has certain limitations. Our model relies solely on TCGA-derived data, which may introduce cohort bias. These limitations underscore the necessity of external validation. Additionally, although L–R pairs provide mechanistic insights, their causal roles require functional experimental validation. Conducting prospective clinical trials to validate L–R-targeted therapies will facilitate the transition of the model from prognostic assessment to therapeutic application.

## 5. Conclusions

In conclusion, we established a robust prognostic model driven by four key ligand–receptor axes, validated across single-cell and spatial dimensions to pinpoint their specific cellular niches. By integrating these molecular insights with a deep learning framework applied to standard H&E slides, we successfully linked high-risk microenvironmental crosstalk to interpretable histological phenotypes. This multi-modal convergence of interactomics and pathomics offers a powerful and methodological framework for risk stratification.

## Data Availability

The datasets generated during and/or analyzed during the current study are not publicly available, but are available from the corresponding author on reasonable request.

## Supplementary Materials

The following supporting information can be downloaded at: https://www.mdpi.com/article/10.3390/biomedicines14051110/s1, Figure S1. The flowchart of the research and data processing. Figure S2. Forest plot summarizing the hazard ratios (HR) of cancer prognosis-associated ligand–receptor pairs. The red dots represent the HR values. Figure S3. LASSO coefficient profiles of the candidate L–R pairs. Each colored line represents a distinct L–R interaction variable. Figure S4. Bar chart displaying the variable importance scores of different L–R pairs, indicating their contribution to the accuracy of the Random Forest model. Figure S5. Bar plot showing genes with significantly different mutation frequencies between the high- and low-risk groups. Figure S6. Box plots comparing the estimated drug sensitivity (IC50 values) of potential therapeutic agents between high- and low-risk patients. Figure S7. Cell type annotation and marker gene expression. The dot plot visualizes the expression of cell type-specific markers. The x-axis represents marker genes, and the y-axis represents cell clusters. Dot size corresponds to the percentage of cells expressing the gene, and color intensity (red) indicates the average expression level.
