# Supplementary material for: Ligand–Receptor Interaction Combined with Histopathology Improves Glioma Prognostic Model
Source: Biomedicines. 2026 May 14;14(5):1110. doi: 10.3390/biomedicines14051110 (PMC13204305; doi:10.3390/biomedicines14051110)
Supplement: Supplementary file 1 [file biomedicines-14-01110-s001.zip › FigS4_Variable_Importance.pdf]

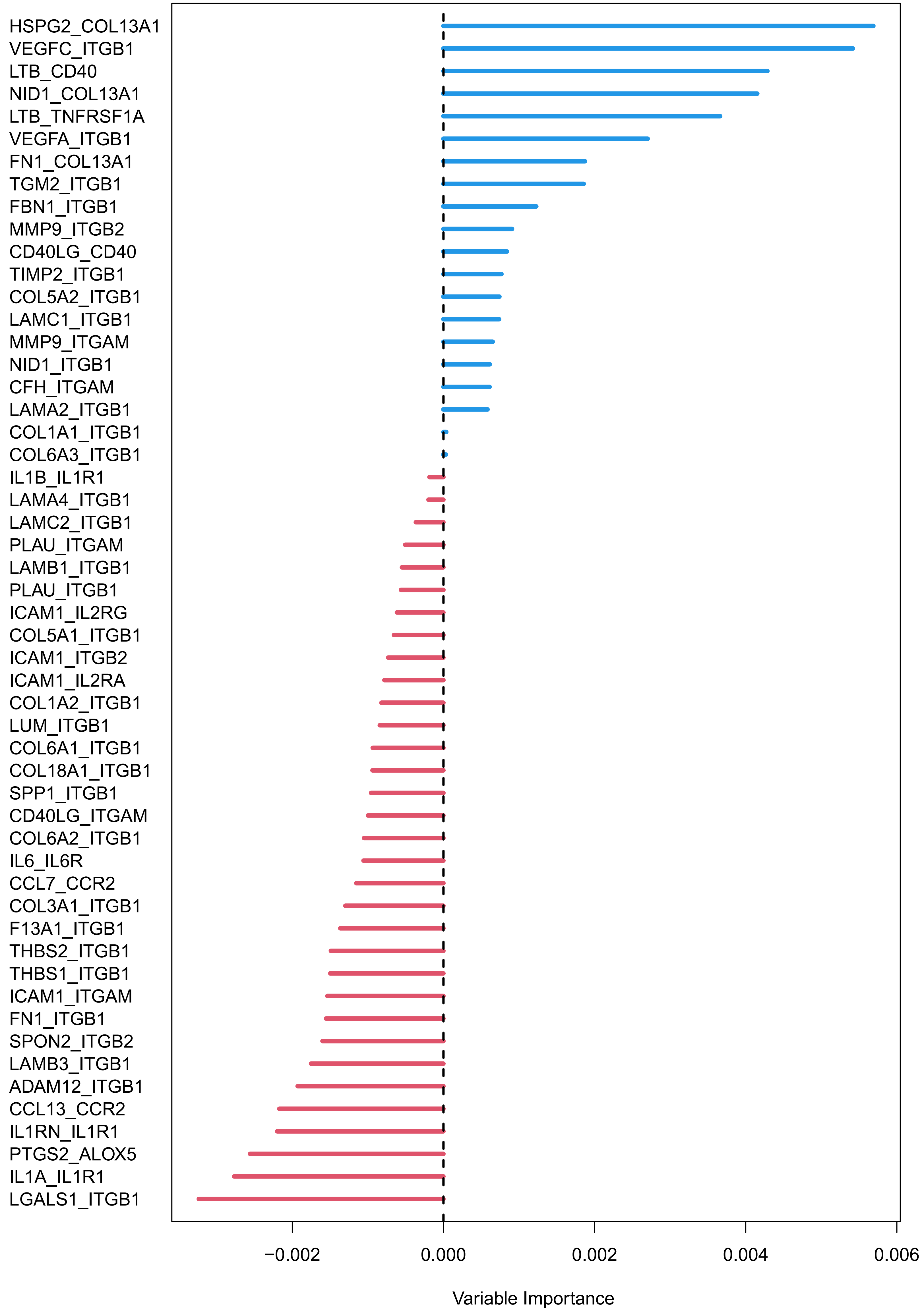
HSPG2_COL13A1
VEGFC_ITGB1
LTB_CD40
NID1_COL13A1
LTB_TNFRSF1A
VEGFA_ITGB1
FN1_COL13A1
TGM2_ITGB1
FBN1_ITGB1
MMP9_ITGB2
CD40LG_CD40
TIMP2_ITGB1
COL5A2_ITGB1
LAMC1_ITGB1
MMP9_ITGAM
NID1_ITGB1
CFH_ITGAM
LAMA2_ITGB1
COL1A1_ITGB1
COL6A3_ITGB1
IL1B_IL1R1
LAMA4_ITGB1
LAMC2_ITGB1
PLAU_ITGAM
LAMB1_ITGB1
PLAU_ITGB1
ICAM1_IL2RG
COL5A1_ITGB1
ICAM1_ITGB2
ICAM1_IL2RA
COL1A2_ITGB1
LUM_ITGB1
COL6A1_ITGB1
COL18A1_ITGB1
SPP1_ITGB1
CD40LG_ITGAM
COL6A2_ITGB1
IL6_IL6R
CCL7_CCR2
COL3A1_ITGB1
F13A1_ITGB1
THBS2_ITGB1
THBS1_ITGB1
ICAM1_ITGAM
FN1_ITGB1
SPON2_ITGB2
LAMB3_ITGB1
ADAM12_ITGB1
CCL13_CCR2
IL1RN_IL1R1
PTGS2_ALOX5
IL1A_IL1R1
LGALS1_ITGB1
−0.002
0.000
0.002
0.004
0.006
Variable Importance
