# Supplementary figures and images for: Ligand–Receptor Interaction Combined with Histopathology Improves Glioma Prognostic Model
Source: Biomedicines. 2026 May 14;14(5):1110. doi: 10.3390/biomedicines14051110 (PMC13204305; doi:10.3390/biomedicines14051110)

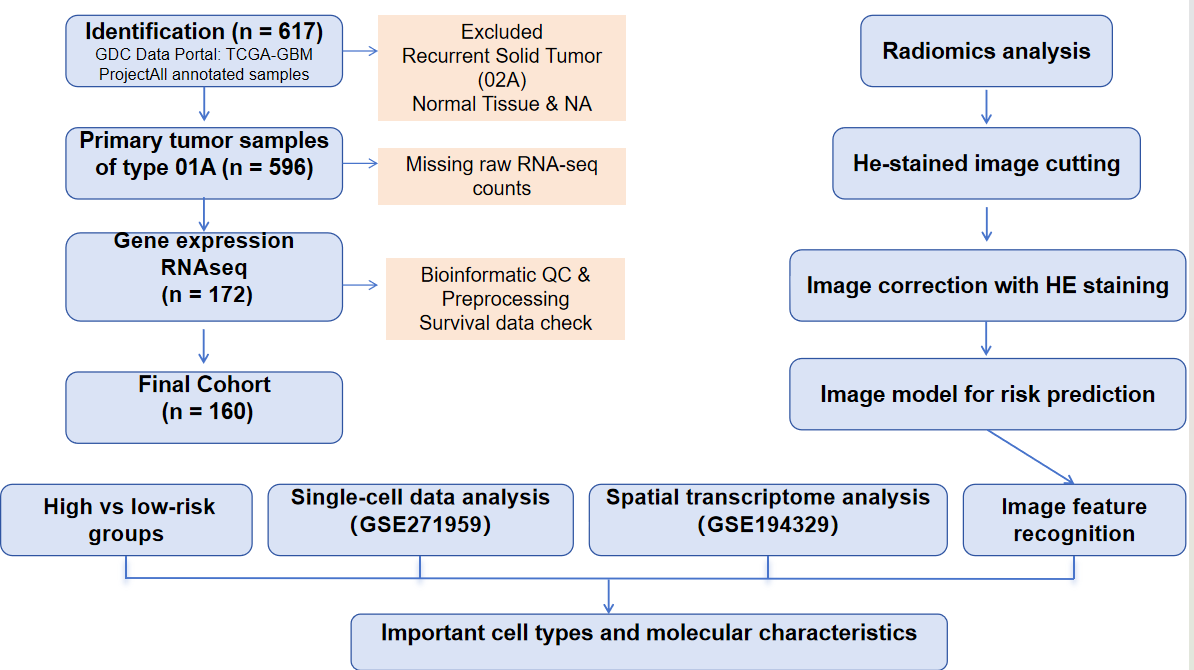

Supplement: Supplementary file 1 [file biomedicines-14-01110-s001.zip › FigS1-workflow.png]

Forest Plot of Prognostic Ligand–Receptor Pairs

Ligand–Receptor Pair

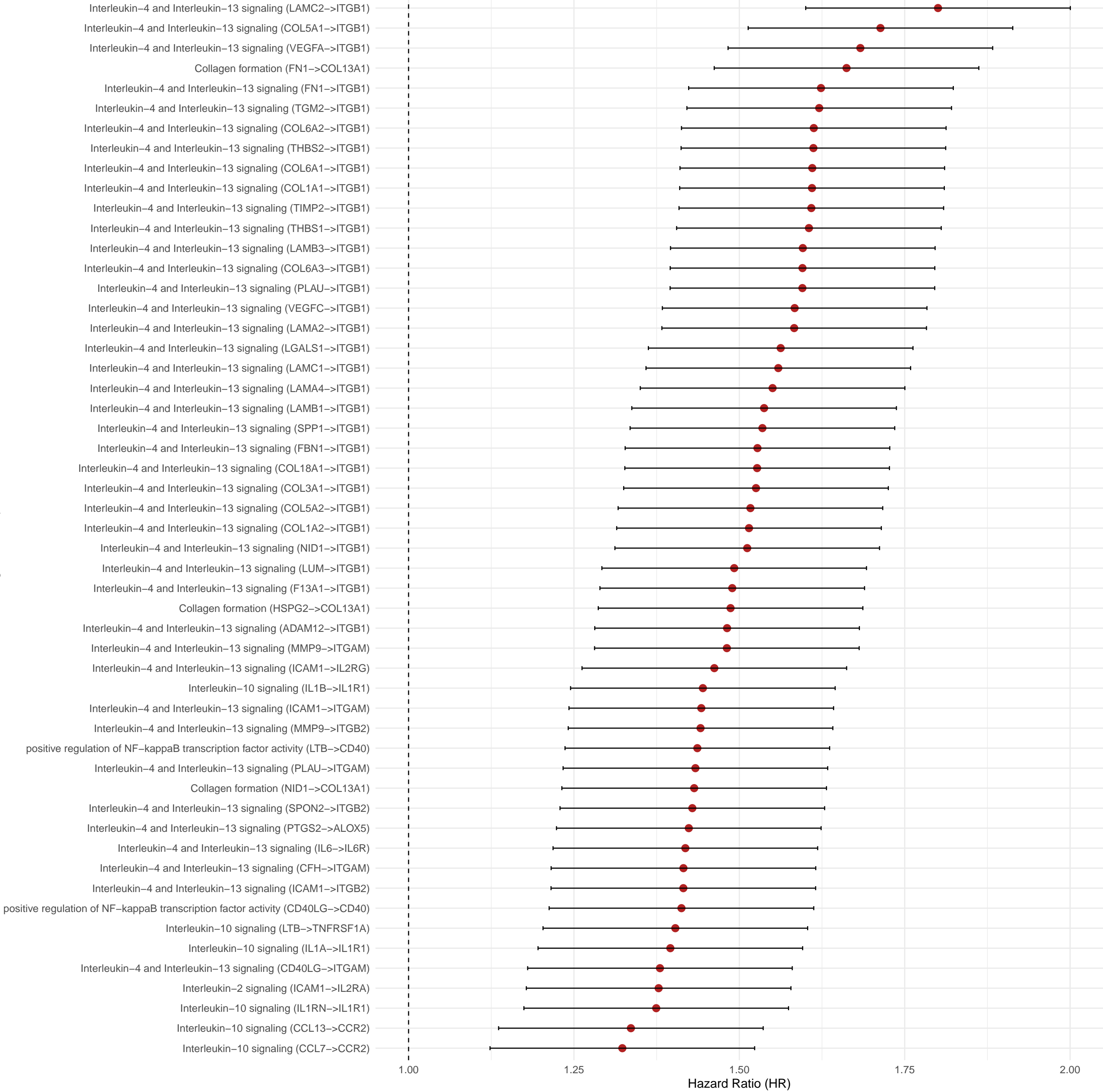

Supplement: Supplementary file 1 [file biomedicines-14-01110-s001.zip › FigS2_forestplot_of_sig_survival_related_pairs.pdf]

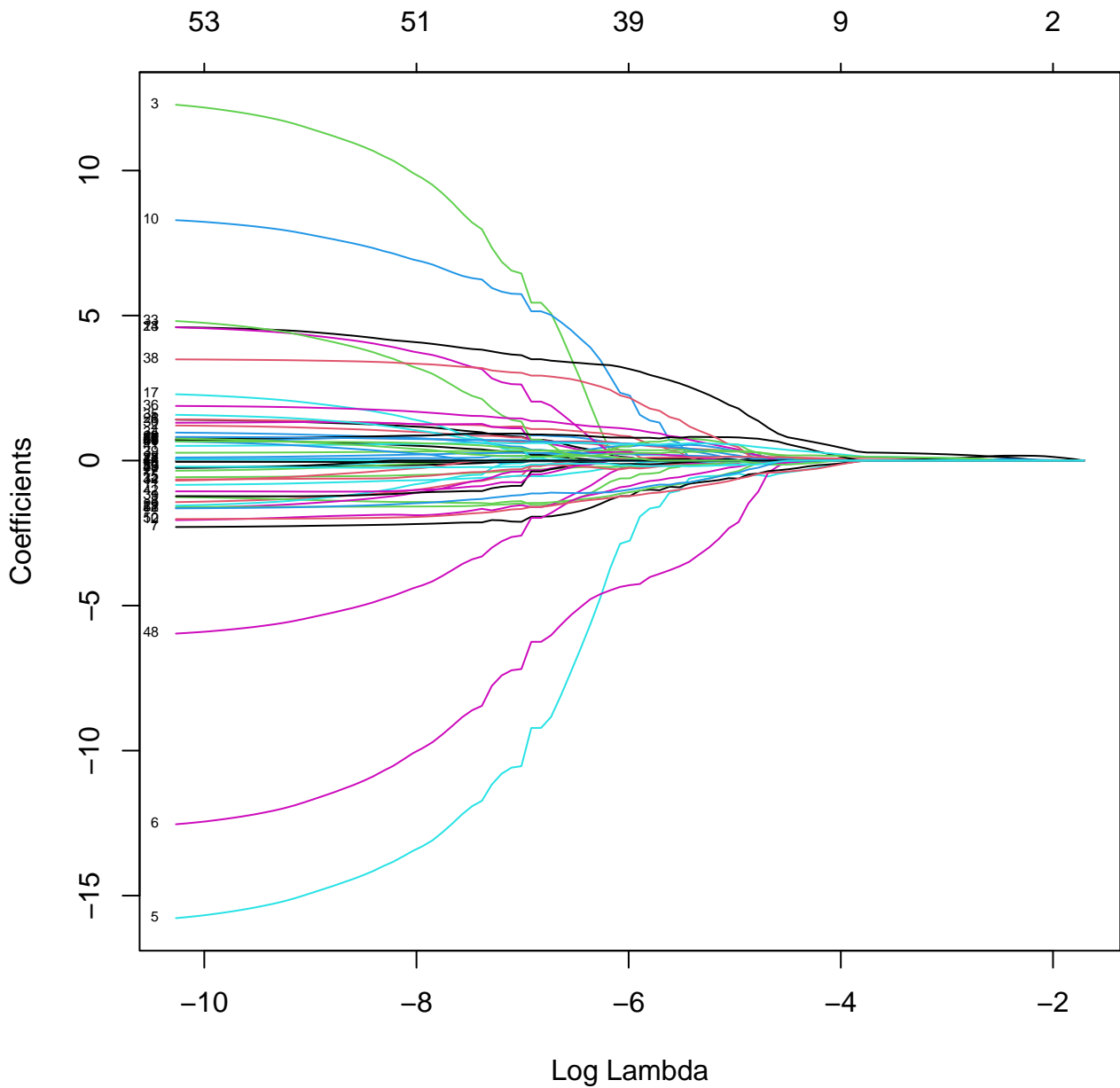

Supplement: Supplementary file 1 [file biomedicines-14-01110-s001.zip › FigS3_lasso_glmnet.fit.pdf]

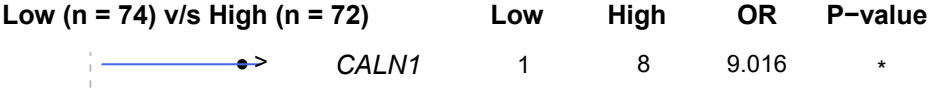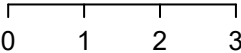

Odds ratio with 95% CI  
(1 = no effect, < 1 Low has more mutants)

Supplement: Supplementary file 1 [file biomedicines-14-01110-s001.zip › FigS5_forestPlot.pdf]

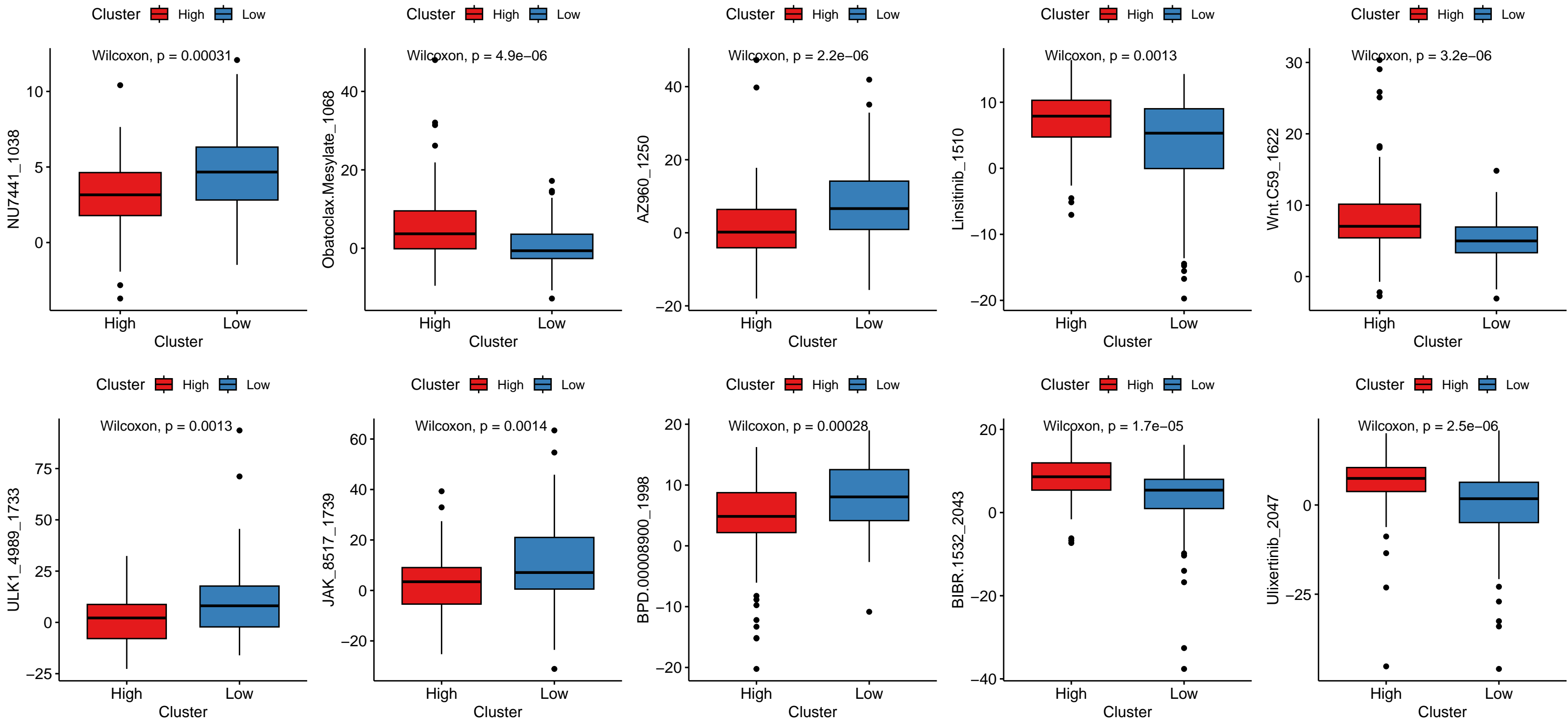

Supplement: Supplementary file 1 [file biomedicines-14-01110-s001.zip › FigS6_Drug_IC50.pdf]

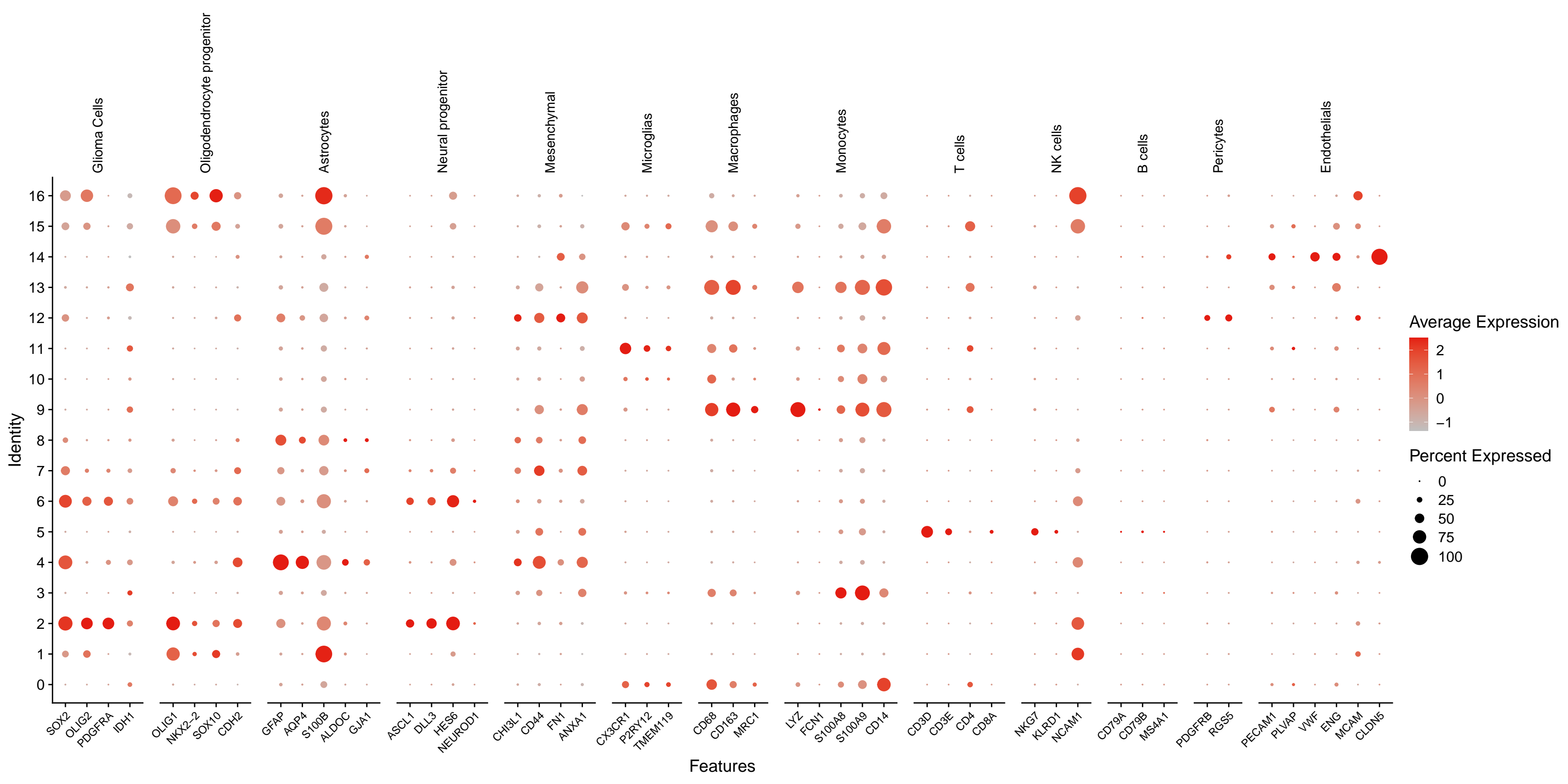

Supplement: Supplementary file 1 [file biomedicines-14-01110-s001.zip › FigS7_feature.DotPlot.pdf]
